# The Integrating Cultural Aspects Into Diabetes Education (INCLUDE) Study to Prevent Diabetes in Chinese Immigrants: Protocol for a Randomized Controlled Trial

**DOI:** 10.2196/65455

**Published:** 2024-11-19

**Authors:** Lu Hu, Nelson F Lin, Yun Shi, Jiepin Cao, Mary Ann Sevick, Huilin Li, Jeannette M Beasley, Natalie Levy, Kosuke Tamura, Xinyi Xu, Yulin Jiang, Iris Ong, Ximin Yang, Yujie Bai, Liwen Su, Sze Wan Chan, Stella S Yi

**Affiliations:** 1 Department of Population Health NYU Grossman School of Medicine New York, NY United States; 2 Center for Healthful Behavior Change, Institute for Excellence in Health Equity NYU Grossman School of Medicine New York, NY United States; 3 UC Berkeley–UCSF Joint Medical Program Berkeley, CA United States; 4 Department of Medicine NYU Grossman School of Medicine New York, NY United States; 5 Department of Nutrition and Food Studies Steinhardt School of Culture, Education, and Human Development New York University New York, NY United States; 6 Socio-Spatial Determinants of Health (SSDH) Laboratory, Population and Community Health Sciences Branch, Division of Intramural Research National Institute on Minority Health and Health Disparities National Institutes of Health Bethesda, MD United States

**Keywords:** mHealth, mobile health, prediabetes, immigrants, prevention, diabetes, INCLUDE, T2DM, social determinants of health

## Abstract

**Background:**

Type 2 diabetes (T2D) contributes to significant morbidity and mortality for Chinese immigrants in the United States, exacerbated by social determinants of health (SDOH) barriers such as language barriers, limited access to healthy foods, and low health literacy.

**Objective:**

The goal of the Integrating Cultural Aspects into Diabetes Education (INCLUDE) study is to test a social media–based intervention adapting the Diabetes Prevention Program (DPP) for Chinese immigrants alongside a culturally adapted, community-supported agriculture program. Here, we report the protocol for the INCLUDE study.

**Methods:**

INCLUDE is a 3-year randomized controlled trial (n=150). Participants with prediabetes or at risk for T2D are enrolled and randomized into either the control or intervention group (n=75 each). Participants from the intervention group receive 2-3 culturally tailored, in-language DPP videos weekly for 12 weeks, as well as biweekly phone calls from bilingual study staff to review video content, support goal setting, and assess and address SDOH-related barriers such as food insecurity. Intervention participants will also be given produce for 10 weeks as part of the community-supported agriculture program. Weight (primary outcome), self-efficacy, diet, physical activity, and food insecurity (secondary outcomes) are measured at baseline, 3-month, and 6-month intervals. Splined linear mixed models will be used to examine group differences in longitudinal weight and other secondary outcomes. The INCLUDE study was approved by the Institutional Review Board at the NYU Grossman School of Medicine.

**Results:**

Recruitment started in May 2023, with the first cohort of 75 participants enrolled and randomized into 2 groups in July 2023. The 3-month and 6-month assessment of the first-year cohort has been completed. We have recruited 75 participants for the second cohort as of July 2024.

**Conclusions:**

The INCLUDE study will serve as an innovative model for culturally adapted, multilevel interventions for underserved communities previously unable to access evidence-based diabetes prevention initiatives. Aligning with several national calls for multilevel interventions, the INCLUDE intervention will provide critical data that will inform how researchers and public health professionals address SDOH barriers faced by underserved populations and prevent diabetes.

**Trial Registration:**

ClinicalTrials.gov NCT05492916; https://clinicaltrials.gov/study/NCT05492916

**International Registered Report Identifier (IRRID):**

DERR1-10.2196/65455

## Introduction

Diabetes poses a significant public health burden. Recent estimates suggest that, in 2021, more than 38 million people in the United States were living with the disease [1]. Type 2 diabetes (T2D) is the most common form of the disease, affecting 90% to 95% of those with diabetes and contributing to significant morbidity and mortality [2]. Compared with those without diabetes, people with diabetes demonstrate mortality rates that are 2 to 3 times higher and a life expectancy that is reduced by as many as 8 years [3,4]. Previous research has also identified a disproportionate burden of T2D among Asian Americans, who have the highest rate of undiagnosed diabetes (51%) among all racial and ethnic groups [5]. Moreover, Asian Americans underutilize health care services [6] and, in the wake of the COVID-19 pandemic, demonstrated the sharpest decline in screening and preventive care among all racial or ethnic groups [7]. Trends such as these point to the need for feasible approaches to address diabetes disparities observed in Asian Americans in the community setting.

As the largest Asian and Pacific Islander subgroup, Chinese immigrants bear high T2D and prediabetes burdens, particularly in New York City (NYC), where almost 1 in every 2 Chinese adults has T2D or prediabetes [8,9]. Additionally, 18% of Chinese adults in NYC who are aged 18 years and older live in poverty; this burden is particularly pronounced among those aged 65 years and older, where 30% live in poverty [10]. This burden of poverty is compounded by other social determinants of health (SDOH) barriers in Chinese immigrant communities, including high rates of limited English proficiency (LEP), lack of health insurance, and poor access to health care [11,12].

The Diabetes Prevention Program (DPP) is an evidence-based intervention to prevent or delay T2D [13]. However, many SDOH barriers limit the access of DPP to underserved Chinese immigrants, contributing to worse diabetes outcomes in Chinese immigrants with T2D compared with non-Hispanic White adults [14]. Differing cultural norms such as food representations (eg, rice vs bread as a major carbohydrate source) [15] also complicate the effective delivery of DPP following the Centers for Disease Control and Prevention’s curriculum without adaptation [12,16]. Furthermore, there is a significant shortage of cultural- and linguistic-concordant providers to deliver the DPP. These barriers are further amplified by the fact that the DPP is often delivered via multiple in-person visits, which can be challenging for Chinese immigrants with LEP who often have long working hours and limited sick time [11,17].

High social media use among Chinese communities, particularly on the free and popular social media app WeChat (Tencent), suggests a promising mechanism for enhancing access to the DPP [18-20]. If shown to be efficacious, culturally and linguistically tailored DPP videos that can be widely disseminated using a free social media platform could be a transformative new model to enhance access to diabetes education and promote health equity for underserved immigrant and minority groups with LEP. This approach can address many barriers for low-income immigrant participants with LEP to accessing DPPs (eg, language and cultural barriers; transportation challenges; lack of sick time; and competing work, childcare, or family obligations) [11,12]. In earlier work, we developed 24 culturally and linguistically tailored diabetes management videos and delivered them to 30 Chinese immigrants with LEP via WeChat, successfully demonstrating feasibility, satisfaction, and potential efficacy in reducing hemoglobin A_1c_ (HbA_1c_) and body weight in Chinese immigrants with existing T2D [18].

A mobile health (mHealth) intervention that leverages social media may have unprecedented advantages for increasing access to the DPP and holds strong promise for scalability. Yet, mHealth alone cannot address the many SDOH barriers to care [21]. According to the National Institute on Minority Health and Health Disparities Research Framework, multilevel interventions are needed to address SDOH barriers in populations with health disparity [22]. One particular issue of an area where this is the case is food insecurity and lack of access to fruits and vegetables, a major concern for Chinese immigrants with LEP [23]. Community-supported agriculture (CSA) programs, which involve a community of individuals who support a farm and in return receive distributions of the farm’s produce throughout the growing season, have been demonstrated to be an effective way to improve food security in White populations and may help to address food access issues in Chinese immigrants with LEP [24,25]. Our recent pilot among 38 participants of a culturally appropriate CSA featuring Chinese produce and flexible payment structures increased vegetable intake (self-report and biomarker) and the variety of vegetables consumed [26]. As a result, we seek to adapt an existing T2D management intervention to include video content relevant to the DPP combined with a culturally appropriate CSA model, taking a multilevel approach to diabetes prevention for Chinese immigrant populations.

The Integrating Cultural Aspects into Diabetes Education (INCLUDE) study is a randomized controlled trial (RCT) within Chinese immigrants at risk of developing T2D. We aim to test a social media–based DPP video program combined with a culturally appropriate CSA program, both tailored for Chinese immigrants. The primary objective is to measure the efficacy of the INCLUDE intervention on body weight; in addition, patient-centered secondary outcomes and qualitative understandings of both facilitators and barriers to program implementation and intervention delivery will be explored. This report describes the INCLUDE study protocol.

## Methods

### Study Design

The proposed study is a 2-arm RCT. Participants are randomized equally to one of two arms (n=75 each): (1) waitlist control or (2) 12-week INCLUDE intervention. Measurements occur at baseline, 3 months, and 6 months. The waitlist control was chosen since we want to provide the INCLUDE intervention to the control group at the end of the study for health equity considerations. We followed the SBIRT (Screening, Brief Intervention and Referral to Treatment) guideline in reporting the guideline. A complete SBIRT checklist is available in Multimedia Appendix 1.

### Ethical Considerations

This study was approved by the NYU Grossman School of Medicine Institutional Review Board (protocol 22-00783) and was registered at ClinicalTrials.gov (NCT05492916). The study adheres to the principles of the Declaration of Helsinki and complies with all relevant privacy and confidentiality regulations regarding participant data. Written informed consent was obtained from all participants prior to data collection. All data will be de-identified before analysis to protect participant confidentiality.

### Eligibility Criteria

To be eligible for the study, participants must (1) self-identify as a Chinese immigrant or Chinese American; (2) be between 18 and 70 years old; (3) have a medical diagnosis of prediabetes or have a prediabetes risk test score [27] of greater or equal to 5; (4) have a BMI ≥23 kg/m^2^ (overweight criteria for Asian individuals) [28]; (5) be willing to receive brief videos regarding diabetes prevention; and (6) possess a smartphone or, if they do not have one, be willing and able to use a study smartphone.

Individuals are excluded from participation if they meet any of the following: (1) unable or unwilling to provide informed consent; (2) unable to participate meaningfully in the intervention (eg, uncorrected sight and hearing impairment); (3) unwilling to accept randomization assignment; or (4) are pregnant, plan to become pregnant in the next 6 months or during the study, or are breastfeeding.

### Recruitment

#### Overview

We will enroll 150 Chinese immigrants who are at risk for T2D, drawing participants from a wide array of community-based organizations and health care facilities serving the Chinese community throughout NYC. Additionally, we recruit participants from several community-based programs embedded in the NYU Langone Health systems like the Table Food Pantry and Together Growing Strong, which provide programming for underserved families in the Sunset Park neighborhood of Brooklyn, NYC.

This RCT is a 3-year study funded by the American Diabetes Association from July 2022 to June 2025.

#### Recruitment Strategies

This study uses multipronged recruitment strategies. We have formed a community advisory board, which includes 5 community-based organizations who have a long history of serving low-income Chinese American communities in NYC. The community advisory board provided important feedback on how to conduct outreach and engage the target population, including holding a press release, inviting local ethnic newspaper and media outlets, community events, and social media advertisement. Posters in simplified Chinese are placed at these community-based organizations and distributed during Chinese community events, in newspapers, or on social media to promote self-referrals. Recruitment materials list a contact telephone number that interested participants can call to reach bilingual staff members who speak Mandarin, Cantonese, and English or a QR code that they can scan.

Additionally, through NYU Langone Health DataCore, we use the Epic electronic medical record (Epic Systems Corporation) to generate a report of potentially eligible participants from across the NYU Langone Manhattan, Queens, Brooklyn, and Long Island campuses. The report includes demographics including name, race, ethnicity, preferred language, gender, date of birth, address, phone number, weight, height, BMI, name of primary care physician, and date of measurement. A letter in both English and simplified Chinese (front and back) describing the study, its goals, and information on how to participate are mailed to eligible participants. About 2 weeks after mailing out the letter, the study team follows up with a phone call to provide more information about the study and assess interest from potentially eligible participants.

#### Randomization and Intervention Groups

##### Screening

Potential participants are contacted by NYU bilingual study staff via phone call and provided with detailed information about the study. If participants express interest in participating, the study staff screen for eligibility by phone. Once eligibility is confirmed, staff will send participants a copy of the key information sheet in English and Chinese and schedule them for baseline data collection. Before conducting the baseline assessment, the study staff will obtain informed consent either through verbal consent via phone call or written consent through in-person sessions. The baseline data collection is conducted either in-person or via phone call. Participants will complete baseline survey consisting of self-report questionnaires with support from bilingual study staff in their preferred language (Mandarin, Cantonese, and English).

##### Randomization

The study biostatistician uses stratified randomization to assign participants. The matching of gender and age will be implemented when participants are randomly assigned to either the control group or the INCLUDE intervention group.

##### Intervention Description

Our mHealth intervention builds on the DPP developed by the DPP Research Group, which significantly decreased the incidence of T2D in a majority female and White cohort [13]. The DPP is primarily based on Western diet and culture and delivered in English, which has limited reach among Chinese immigrants with LEP due to language and culture barriers. Our team has culturally and linguistically adapted the DPP and developed brief videos tailored to low-income Chinese immigrants. Our video-based DPP is based on Social Cognitive Theory, the idea that self-efficacy shapes behavior and performance. Self-efficacy is influenced by 4 sources of information including mastery experiences, social modeling, verbal persuasion, and physiological states [29]. As an example of enhancing mastery experience, our intervention videos encourage participants to set incremental and achievable goals and reward themselves for reaching these goals. Further, our DPP videos address behavioral triggers, lapses, and relapses that may inhibit self-management. Our intervention also uses social modeling by featuring a Chinese immigrant community member in videos who was able to prevent or delay the onset of T2D. We also have videos guiding participants to recognize physiological states associated with diabetes prevention efforts, such as reduced body weight.

Closely aligned with the DPP curriculum, our videos cover topics from diabetes, prediabetes, nutrition, healthy eating, physical activity, stress management, sleep, and behavioral techniques (eg, goal setting, problem-solving, and social support; Table 1). These videos are designed to provide participants with important education and skills and empower them to make behavioral changes. All the videos are in Mandarin Chinese, tailored for Chinese culture and norms (eg, Chinese diet, cooking methods, recipes, cultural beliefs, and healthy eating during Chinese holidays). The videos are created with short sentences and language for lay audiences to accommodate limited health literacy reported in low-income Chinese immigrants.

**Table 1:** Intervention content.

Week	Content
1	Program overview and introducing the team, diabetes and prediabetes, goal setting
2	Get active to prevent T2D^a^, track your activity, eat well to prevent T2D
3	Track your food and read the label, get more active
4	Burn more calorie than you take in, shop to prevent T2D, cook to prevent T2D
5	Manage stress, find time for fitness, cope with triggers
6	Keep your heart healthy, Take charge of your thoughts
7	Get support, at well away from home, stay motivated to prevent T2D
8	When weight loss stalls, take a fitness break, stay active to prevent diabetes
9	Stay active away from home, more about T2D
10	More about carbs, have the healthy food you enjoy, attending doctors’ appointments
11	Navigating the US health care system, get enough sleep
12	Get back on track, prevent T2D for Life

^a^T2D: type 2 diabetes.

**Figure 1:**
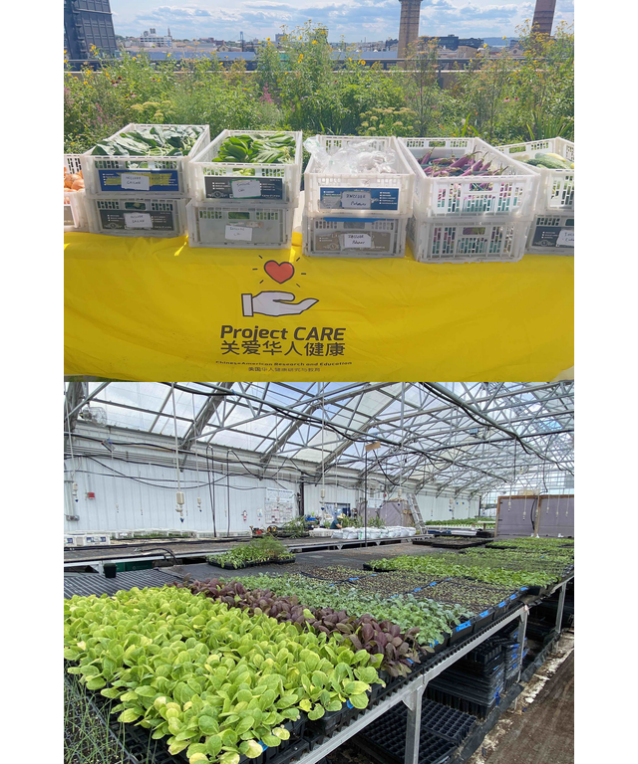
Fresh produce pick-up.

In addition to the educational videos, our intervention provides fresh produce to the participants via a CSA program. Bilingual study staff coordinate between the rooftop urban community farm which runs the CSA, Brooklyn Grange, and study participants. Participants receive weekly fresh and culturally appropriate fresh produce every Tuesday for 10 weeks. Each week, participants will be offered 6-7 different varieties of fresh produce, with a total weekly value of US $25. Examples of produce provided include gai lan, bok choy, and sword lettuce (Figure 1).

Participants in the INCLUDE group receive standard care alongside brief DPP videos and fresh produce from the CSA program. We leverage the free and popular social media platform, WeChat, to deliver DPP videos. We send 2-3 DPP videos each week for a total of 12 weeks with each video lasting about 5 minutes in duration. Uptake of videos is further supported by biweekly phone calls from study staff trained in nutrition to review and answer any questions about the video content and support participants in both setting and reaching goals around diabetes prevention. In addition, participants are given produce for 10 weeks as part of the CSA program. Staff help participants join the CSA program and assess and address other SDOH barriers such as food insecurity.

##### Waitlist Control Group

Control participants continue to receive the standard of usual care with their own providers during our study. At the end of the study, the control group is provided the opportunity to receive DPP videos and gift cards with a value equal to the 10-week CSA program.

#### Procedures

A steering committee, comprised of the study principal investigator, coinvestigators, and study team, is overseeing the trial implementation. The study includes measurement visits at baseline, 3 months, and 6 months. Survey data are collected in language over the phone or in person at community site offices. After each measurement call is completed, participants are paid US $25. Further, at 6 months (the end of the program) the year-1 control group are paid US $250, the cash value of the CSA program received by the intervention group. All incentive payments are provided through ClinCard, which are provided to them at baseline visits or mailed to their home address.

Based on our initial experiences working with intervention group participants, we will develop focus group guides to characterize barriers to and facilitators of program participation and intervention delivery. We will conduct focus groups with participants, community-based organizations, study staff to gain a deeper understanding of barriers to and facilitators of program participation and intervention delivery. These are informed by the Consolidated Framework for Implementation Research (CFIR) [30], which posits five key domains affect intervention outcomes, including (1) the characteristics of the intervention, (2) the inner setting of the organization, (3) the outer setting of the organization, (4) individuals involved in the intervention, and (5) the implementation process.

We will conduct 2 participant focus groups in Chinese (n=5-8 participants each), with one group demonstrating high engagement with the INCLUDE intervention (eg, they watch at least 70% of the DPP videos or at least 70% participation rate in the CSA) and another group demonstrating low engagement (eg, watch less than 70% of the DPP videos or less than 70% participation rate in the CSA program) to understand beliefs and attitudes around diabetes prevention, perceptions of the INCLUDE program, challenges encountered, and recommendations for improving the intervention.

We will also conduct focus groups with community partners (n=5-10) to understand perceptions of the INCLUDE program’s effectiveness, factors that facilitated or blocked implementation, and perceptions of their role in the INCLUDE program.

Finally, we will conduct focus groups with study staff to understand barriers and facilitators of the INCLUDE program implementation.

#### Measures

##### Primary Outcome

Weight is obtained using an electronic scale. Scales are mailed to the participants’ homes. For baseline weight, most of the year-1 participants were able to attend in-person baseline measurement visit and were instructed to step on the study scale with light clothing. For 3- and 6-month follow-up data, year-1 participants completed phone-based survey and were instructed to step on the study provided scale at home and reported back the weight data.

##### Secondary Outcomes

As secondary outcomes, we will measure self-efficacy, dietary intake, physical activity, and food insecurity at each time point (baseline, 3 months, and 6 months).

##### Self-Efficacy

We will use the well-validated Weight Efficacy Lifestyle Questionnaire [31] to measure participants’ confidence in resisting eating in various situations. A higher score indicates a higher level of self-efficacy. This instrument contains 20 items and asks participants to rate their confidence level in resisting eating, using a 10-point Likert scale ranging from 0 (not confident) to 9 (very confident).

##### Dietary Intake

Informed by a previous study and our own studies in Chinese Americans [19,32], we will use the 8-item Starting the Conversation diet scale [33] to assess dietary intake.

##### Physical Activity

We will use the International Physical Activity Questionnaire [34] short version to assess the frequency and duration of various physical activities undertaken by adults over the past 7 days. This scale provides an estimate of the number of minutes per week participants engage in each physical activity intensity (ie, sedentary, light, moderate, and vigorous).

##### Food Insecurity

We will use the 18-item San Francisco Chinese Food Security Module [35] to assess food access and insecurity in Chinese immigrants. This scale was culturally and linguistically adapted from the US Household Food Security Survey Module [36] and has been pilot-tested and validated with Chinese immigrant community members and professionals from clinical settings [35].

#### Covariates

We will use a sociodemographic and health questionnaire to collect basic information about participants such as age, gender, education, income, duration of residence in the United States, English proficiency, duration of prediabetes, and medical history. We also use a scale adapted from the Short Acculturation Scale for Hispanics [37] to evaluate various levels of acculturation in the sample and adjust for this in the statistical models if needed. The adapted scale has established reliability and validity in Chinese immigrants [38]. We will use the SDOH (Core) survey [39] from the National Institute of Health PhenX Toolkit to assess SDOH in Chinese immigrants.

#### Data Analysis

An “intent-to-treat” approach will be used to address the aims of our study regarding weight change and secondary health outcomes. A linear mixed model will be used to test time-specific differences in both primary and secondary outcomes attributable to the intervention. Randomization should obviate the need for adjustment but, in the case of unbalanced baseline covariates or for the variables that are anticipated to be associated with the outcome, these variables will be adjusted as necessary in the analyses. We will use splined linear mixed models with repeated measures to compare changing trends in different periods: 0-3 months and 3-6 months. In this analysis, we will adjust for the covariates as needed. Data analysts will be blinded to group allocation.

To understand barriers and facilitators of program participation, we will use NVivo software (version 14.0; Lumivero) to conduct content analysis using both deductive (CFIR theory driven) and inductive coding. We will create an initial codebook based on the constructs from the CFIR framework. Two coders will independently code the first 5 transcripts and meet to discuss agreement and disagreement to further refine the initial codebook. The 2 coders will code the remaining transcripts independently. Once coding is complete, the coders and the study team will meet to discuss relationships between codes and further identify subthemes, and themes.

### Sample Size Justification

In our single-group, pre-post test, feasibility study of the diabetes management video intervention [20], we observed a significant 6.37% (95% CI 2.16%-10.58%; *P*=.005) weight loss at 6 months compared with baseline, and the SD of percent weight loss at 6 months was 10.64%. Based on these pilot data, we conservatively estimate a group difference of 5% in weight loss between the control and INCLUDE groups at 6 months; this degree of weight loss is considered clinically significant [28]. Using the observed SD of 10% in our preliminary study, based on a 2-sample, 2-sided *t* test, 64 per group will be required to detect a minimum group difference of 5% in percent weight loss with a power of 80% and a type I error of 0.05. Our prior studies have achieved ~85% to 97% retention. With a conservative retention rate of 85%, we will recruit 150 (75 per group) participants to yield a final sample of at least 128 (64 per group).

## Results

Recruitment started in May 2023, with the first cohort of 75 participants enrolled and randomized into 2 groups in July 2023. The 3-month and 6-month assessment of the first-year cohort has been completed. We have recruited 75 participants for the second cohort as of July 2024. A detailed timeline is provided in Multimedia Appendix 2.

## Discussion

### Overview

This protocol describes the design for an RCT testing a multilevel diabetes prevention intervention, including culturally and linguistically appropriate diabetes prevention videos delivered through social media and a culturally tailored CSA produce box program, among Chinese immigrants at risk for T2D. A multilevel intervention administered through the popularly used social media platform of WeChat has great potential to support Chinese immigrants’ understanding of diabetes prevention, increasing self-efficacy and promoting behavior change while decreasing the risk of developing T2D. We hypothesize that participants in the INCLUDE intervention group will achieve greater weight loss after the program and have better psychosocial and behavioral outcomes, including self-efficacy, dietary intake, physical activity, and food security than the control group.

Access to culturally tailored DPP programs is a significant public health concern for immigrants with LEP, racial and ethnic minority groups, and rural populations in the United States [40]. This project will serve as an innovative model to mitigate access issues for underserved populations. Aligning with several national calls for multilevel interventions, the INCLUDE study will provide critical data on the effect of a multilevel intervention to address SDOH barriers faced by underserved populations. This study may have important implications for other underserved immigrant populations (eg, Latino immigrants or South Asian immigrants) who also experience high chronic disease burden, face numerous SDOH barriers, and frequently use social media like WhatsApp [41].

### Strengths, Limitations, and Future Directions

One major limitation of the INCLUDE intervention is that videos were only developed in Mandarin Chinese. For Chinese immigrants who only speak regionalized dialects such as Cantonese, Fujianese, or Fuzhounese but not Mandarin, this poses a barrier to understanding intervention content. However, given that Mandarin is increasingly a lingua franca spoken by most Chinese immigrants [42], our videos are still accessible to many. Further, while our sample is recruited from the NYC metropolitan area, which has a large ethnic Chinese population in the United States [43], our results may not be generalizable to other areas of the country where public health programming may differ for Chinese immigrant populations.

Despite these limitations, this study will generate important results and insights into the efficacy of multilevel, evidence-based, and culturally and linguistically concordant approaches to diabetes prevention in low-income, immigrant populations. These results will inform the development of future mHealth and multilevel interventions that target other immigrant communities facing disparate T2D burdens as well as future diabetes prevention programming for Chinese immigrant communities.

### Dissemination Plan

Research findings will be presented through a variety of mediums, including both academic and community-facing outlets. These include regional venues such as the annual NYU Langone Annual Health Equity Symposium and the Biennial Asian American, Native Hawaiian, and Pacific Islander Health Conference at NYU, as well as national meetings such as the American Diabetes Association Annual Symposium. We also anticipate publishing our work in the form of manuscripts in peer-reviewed journals.

Furthermore, we will leverage our relationships with community partners in the Chinese community in NYC to report back to the general public about our findings, as well as potential implications for diabetes prevention in Chinese immigrant communities. Our reporting will take place through town hall forums at community spaces such as the NYU Langone 7th Avenue Family Health Centers or the community-based organizations; one-pagers; infographics for dissemination via WeChat; and short videos describing our research findings delivered in English, Mandarin, and Cantonese.

Chinese immigrants are the second-largest immigrant group in the United States, disproportionately experiencing high T2D and prediabetes burden with poor outcomes. This project aims to provide evidence on strategies to improve access to DPPs for underserved populations. It could also serve as a model for other high-risk immigrant groups with LEP with similar barriers. The study will assess the effectiveness of using an existing social media platform and CSA to improve DPP access. If successful, this approach could inform future scaling efforts and promote health equity among underserved immigrant with LEP and minoritized groups.

## Appendix

Multimedia Appendix 1 SPIRIT (Standard Protocol Items: Recommendations for Interventional Trials) checklist.

Multimedia Appendix 2 Study timeline and measures.

Multimedia Appendix 3 Peer review report from the American Diabetes Association (USA).

## Abbreviations

CSA: community-supported agriculture
CFIR: Consolidated Framework for Implementation Research
DPP: Diabetes Prevention Program
HbA1c: hemoglobin A1c
INCLUDE: Integrating Cultural Aspects into Diabetes Education
LEP: limited English proficiency
mHealth: mobile health
NYC: New York City
RCT: randomized controlled trial
SBIRT: Screening, Brief Intervention and Referral to Treatment
SDOH: social determinants of health
T2D: type 2 diabetes

## Data Availability

The datasets used or analyzed during this study are available from the corresponding author on reasonable request.
