# Supplementary material for: The Integrating Cultural Aspects Into Diabetes Education (INCLUDE) Study to Prevent Diabetes in Chinese Immigrants: Protocol for a Randomized Controlled Trial
Source: JMIR Res Protoc. 2024 Nov 19;13:e65455. doi: 10.2196/65455 (PMC11615548; doi:10.2196/65455)
Supplement: Multimedia Appendix 2 [file resprot_v13i1e65455_app2.docx]

| **Multimedia Appendix 2. Study timeline and measures** | | | | | | |
| --- | --- | --- | --- | --- | --- | --- |
|  | **Study Period** | | | | | |
|  | Enrollment | Allocation | Intervention implementation | | | Close-out |
|  |  |  | Weekly videos | Weekly produce | Biweekly phone call |  |
| **Timepoint** | April to July 2023 (cohort 1)  May to June 2024 (cohort 2) | July 2023 (cohort 1)  July 2024 (cohort 2) | August to October 2023 (cohort1)  August to October 2024 (cohort1) | | | Feb 2024 (cohort 1)  Feb 2025  (cohort 2) |
| Enrollment | X |  |  |  |  |  |
| Eligibility screen | X |  |  |  |  |  |
| Informed consent | X |  |  |  |  |  |
| Allocation |  | X |  |  |  |  |
| **Intervention** |  |  |  |  |  |  |
| Intervention group |  |  | X | X | X |  |
| Waitlist control |  |  |  |  |  | X |
| **Assessment** |  |  |  |  |  |  |
| ***Baseline variables*** |  |  |  |  |  |  |
| Weight | X |  |  |  |  |  |
| Weight Efficacy Lifestyle Questionnaire | X |  |  |  |  |  |
| 8-item Starting the Conversation diet scale | X |  |  |  |  |  |
| The International Physical Activity Questionnaire | X |  |  |  |  |  |
| The 18-item San Francisco Chinese Food Security Module | X |  |  |  |  |  |
| Age, gender, education, income, duration of residence in the US, English proficiency, duration of prediabetes, and medical history | X |  |  |  |  |  |
| The short acculturation scale for Hispanics | X |  |  |  |  |  |
| ***Outcome variables and other measures*** |  |  |  |  |  |  |
| Weight |  |  |  |  |  | X |
| Weight Efficacy Lifestyle Questionnaire |  |  |  |  |  | X |
| 8-item Starting the Conversation diet scale |  |  |  |  |  | X |
| The International Physical Activity Questionnaire |  |  |  |  |  | X |
| The 18-item San Francisco Chinese Food Security Module |  |  |  |  |  | X |
