# Supplementary material for: The Integrating Cultural Aspects Into Diabetes Education (INCLUDE) Study to Prevent Diabetes in Chinese Immigrants: Protocol for a Randomized Controlled Trial
Source: JMIR Res Protoc. 2024 Nov 19;13:e65455. doi: 10.2196/65455 (PMC11615548; doi:10.2196/65455)
Supplement: Multimedia Appendix 3 [file resprot_v13i1e65455_app3.pdf]

Hu, Lu

From: Grantquestions <Grantquestions@diabetes.org>
Sent: Monday, June 6, 2022 3:13 PM
To: Hu, Lu
Subject: ADA Grant Application: Reviewer Critiques

[EXTERNAL]

Dear Dr. Hu,

Thank you for your recent grant application to the American Diabetes Association. As one of ADA's grant review best practices and to ensure full transparency, we are providing you with the external reviewer comments below related to your submission, Grant ID# 7-22-ICTSN-08, titled, "Integrating cultural aspects into diabetes education (INCLUDE)".

On behalf of the American Diabetes Association, thank you for your efforts to advance our mission to prevent and cure diabetes and to improve the lives of all people affected by diabetes. Should you have questions, please don't hesitate to contact Association staff at grantquestions@diabetes.org.

Reviewer 1 Feedback

What is the overall hypothesis the proposal seeks to address?
This proposal seeks to address the health disparity in diabetes prevention in Chinese migrants through a combination of individual and community approaches. The proposal hypothesised that the intervention (INCLUDE) would result in greater weight loss and greater improvements in secondary outcomes including self-efficacy, dietary intake, physical activity and food insecurity.

What are the major strengths and weaknesses of the proposal?
A major strength of this research is the addressing of a significant health gap/ need in diabetes prevention given the high burden of T2D and underserving of this migrant group. The team's track record in this area is also a strength having developed 24 culturally and linguistically tailored video piloted in a feasibility study, and currently addressing the newly discovered need of food insecurity through a community aspect of the intervention. The team also has piloted the community aspect in a feasibility study. The team has demonstrated expertise and feasibility to deliver mHealth interventions.

How could this proposed research be improved? Please be specific.
More evidence/information on the effectiveness of the CSA aspect on improving health outcomes would strengthen this proposal as it is a novel aspect within this intervention. Besides sex, there may be other determinants for differential effect e.g. English literacy, social capacity etc, that could also be considered to increase knowledge in this area.

Is the proposed research being addressed by others?
To my knowledge there is no other similar project using a WeChat+agriculture approach for diabetes prevention in Chinese immigrants

If successful, what would be the potential impact of the proposed research? Ex: how will this improve lives of patients living with diabetes?
If successful, this project could improve diabetes prevention among Chinese immigrants and narrow the health gap between this group and mainstream population

Will the experimental approaches/proposed methods allow for a clear conclusion?

The approach is targeted at behaviour changes and weight loss, allowing for a clear conclusion relevant to diabetes prevention.

Can this work be accomplished within the allocated time frame?
The main concern is setting up the CSA processes and recruitment. The applicant has addressed these concerns. The timeline appears feasible.

Is the proposed research innovative or incremental?
The combination of WeChat-delivered diabetes prevention sessions and community agriculture program is innovative. It addresses barriers identified from the end user group through consumer engagement and pilot studies. The community-aspect is especially novel and could have benefits beyond weight and glycemic changes, such as increasing social capacity and empowerment that could also improve global health outcomes.

Does the applicant have appropriate qualifications to perform the work?
The applicant is qualified to carry out the work. In addition to the teams progress to date described above, Dr Hu has been a PI on projects developing and testing culturally and linguistically tailored mHealth interventions for low-income LEP Chinese immigrants, led pilot awards on this topic, mentored by an experienced team in behavioural methods, community projects, mHealth, nutrition and diet in diverse populations and biostatistics. The team has demonstrate track record in recruiting and retaining Chinese participants.

Are appropriate resources available to the investigator to perform the proposed research (ex: environment, facilities, equipment)?
NYU Langone Health appeared to be offer the appropriate resources for this work. It has an established mHealth unit, fundings and a community advisory board.

Do you have any ethical concerns about the proposed study? If yes, please describe your concerns in detail. If no, please enter N/A.
N/A

Reviewer 2 Feedback

What is the overall hypothesis the proposal seeks to address?
The overall hypothesis is that a mHealth intervention that adapts the diabetes prevention program curricula into videos combined with a CSA program will improve diabetes-related outcomes among low-income Chinese immigrants in Brooklyn, NYC.

What are the major strengths and weaknesses of the proposal?
Strengths:
- The projects builds on preliminary research from the PI (mHealth) and co-I (CSA program) to address multiple level SDOH barriers to improve health of Chinese immigrants
- Three-time point evaluation will allow for longitudinal assessment
- Research team is multidisciplinary and have longstanding collaborations
- Multiple letters of support from community partners, recruitment sites, and Institutional centers
- Inclusion of vegetables and a client-choice model for CSA
- The intervention is based on theory: SCT and NIMHD framework.
Weakness:
- Unclear why weight loss is the main outcome, and not HbA1c. I understand that weight loss is part of diabetes management, but this is not well linked in the proposed.
- Interventions that only promote produce intake have not shown to be as effective as interventions that also discourage intake of unhealthy foods.
- Unclear how participants will continue to get produce from the CSA program once funding ends. How do participants get to the CSA site? I understand that the CSA is located in the same borough where participants get care, but they don't

necessarily live close to one or another to travel weekly for produce. Is COSTCO the main site where participants shop for groceries?
- Sustainability concerns is also related to non-harvest winter months.
- No dissemination plans.

How could this proposed research be improved? Please be specific.
Sustainability of the CSA model could have been discussed. I am interested to hear researchers thoughts on how to maintain CSA participation once funds are over, and how to continue to support access to healthy foods during the winter months. The plan doesn't need to be laid out right now, but qualitative questions to get into sustainability issues could be added for the patient FGD and community partner interviews.
At the end of section D6.2, the proposal states that participants who need additional assistance will be linked to services by the CHW. I think this is a strength of the proposal and perhaps a component that could be better emphasized. Early in the proposal researchers stated that many low-income immigrants were eligible for SNAP, but I would assume that participation rates are low. Connecting participants to SNAP would potentially address the "sustainability" comment and actually impact food insecurity. More information on how the additional assistance will be conducted and where (unclear if in the hospital) would be helpful.

Is the proposed research being addressed by others?
No - this proposal is a combination of previous efforts intervening at the individual and community levels.

If successful, what would be the potential impact of the proposed research? Ex: how will this improve lives of patients living with diabetes?
If successful, this proposal will improve the connection between immigrant and access to health care through technology. This model can be scaled-up and reach more adults with the goal to prevent diabetes.

Will the experimental approaches/proposed methods allow for a clear conclusion?
Yes - the proposed design, intervention, and evaluation would allow researchers to identify whether a DPP delivered via WeChat + a CSA program promotes weight loss and improve healthy behaviors associated with diabetes prevention.

Can this work be accomplished within the allocated time frame?
Timeline is appropriate. Follow-up data collection for those receiving the intervention last might go until Q2, year 3 with possible delays for data analysis.

Is the proposed research innovative or incremental?
The combination of WeChat-delivered diabetes prevention sessions and community agriculture program is innovative. It addresses barriers identified from the end user group through consumer engagement and pilot studies. The community-aspect is especially novel and could have benefits beyond weight and glycemic changes, such as increasing social capacity and empowerment that could also improve global health outcomes.

Does the applicant have appropriate qualifications to perform the work?
The applicant had led various projects with successful data collection and retention, and clear relationship with the community and other organizations. Well qualified to carry out the proposal.

Are appropriate resources available to the investigator to perform the proposed research (ex: environment, facilities, equipment)?
Multiple institutes, centers, and community organizations available and willing to support the research.

Do you have any ethical concerns about the proposed study? If yes, please describe your concerns in detail. If no, please enter N/A.
No.

Reviewer 3 Feedback

What is the overall hypothesis the proposal seeks to address?
The overall hypothesis of the proposal is to adapt a current type 2 diabetes management intervention to incorporate DPP-related video content and a co-located CSA. This intervention, INCLUDE, will be tested against a waist-list control to assess efficacy on both primary and secondary outcomes. Additionally, qualitative data collection via focus groups and interviews will be used to characteristic barriers and facilitators of the intervention.

What are the major strengths and weaknesses of the proposal?
The major strength of the proposal is the clear articulation of a series of research projects that incrementally build upon one another to address the development of a tailored diabetes prevention/management program for Chinese immigrants. The grantsmanship of the application is also a particular strength. The focus on a health disparate population, low-income Chinese immigrants, is also a particular strength.
Could more information be given about what the CONTROL group receives? The application states that the wait-list control will receive the DPP videos or be provided the opportunity to receive the DPP videos, as well as be guided in accessing the CSA. Will these 24 videos be given weekly, similarly to the INCLUDE arm, or at a single time at the end of the study?

How could this proposed research be improved? Please be specific.
How will CSA use in both the INCLUDE and CONTROL arms be assessed?
How will qualitative data and results be triangulated with quantitative data and results?

Is the proposed research being addressed by others?
No, the proposed research does not appear duplicative.

If successful, what would be the potential impact of the proposed research? Ex: how will this improve lives of patients living with diabetes?
If this study is successful, the potential impact would include the development of a culturally congruent diabetes prevention intervention targeting Chinese immigrants who are at risk for type 2 diabetes.

Will the experimental approaches/proposed methods allow for a clear conclusion?
Yes, the research design and methods are well articulated and as described should allow for clear results and conclusions.

Can this work be accomplished within the allocated time frame?
Yes, the applicant provides a nice timeline detailing the completion of the study within the 3 year time period. Additionally, the applications details that recruitment and retention may be an issue, but this should be easily addressed given the solution proposed and the applicant and investigative team's experience with this study population.

Is the proposed research innovative or incremental?
Yes, the proposed research is incremental and builds from a single-arm P&F study to now incorporate additional intervention components and a wait-list control.

Does the applicant have appropriate qualifications to perform the work?
Yes, the applicant/PI is very qualified to perform the work, as demonstrated by their strong track record of extramural funding and resulting publication record.

Are appropriate resources available to the investigator to perform the proposed research (ex: environment, facilities, equipment)?
Yes, appropriate resources are available to the PI and investigative team to performed the proposed research study.

Do you have any ethical concerns about the proposed study? If yes, please describe your concerns in detail. If no, please enter N/A.

No ethical concerns.
